# Exploring a Path Model of Cognitive Impairment, Functional Disability, and Incontinence Among Male Veteran Home Residents in Southern Taiwan

**DOI:** 10.1038/s41598-020-62477-y

**Published:** 2020-03-27

**Authors:** Yung-Yu Su, Ying-Yi Tsai, Chiao-Lee Chu, Chien-Chih Lin, Chun-Min Chen

**Affiliations:** 1 Department of Long-Term Care, National Quemoy University, No. 1, University Rd., Jinning Township, Kinmen County 892 Taiwan (R.O.C.); 2 Kaohsiung Veterans General Hospital Pingtung Branch, No.1. Anping Lane 1. Jausheng Rd., Nei Pu Township, Ping-Tung County 912 Taiwan (R.O.C.); 3 Department of Nursing, Meiho University, No. 23 Ping Kuang Rd., Nei Pu Township, Ping-Tung County 912 Taiwan (R.O.C.); 4 Research Education and Epidemiology Center, Changhua Christian Hospital, No.135 Nan–Hsiao Street, Changhua, 500-06 Taiwan (R.O.C.)

**Keywords:** Risk factors, Disability

## Abstract

Most studies focusing on only one directional effect among cognitive health, physical function, and incontinence may miss potential paths. This study aimed to determine the pathway by analyzing the bidirectional effects of exposure (*X*) on outcome (*Y*) and explore the mediating effect (*M*) between *X* and *Y*. Secondary data analyses were performed in this study. The original data were collected from August to October 2013 in one VH in Tainan, Taiwan, and the final sample size was 144 older male veterans. Path analysis was performed to test the pathway sequence X → M → Y among the three outcome variables. Approximately 80% of the veterans were aged 81 or older, approximately 42% had a functional disability, 26% had cognitive impairment, and 20% had incontinence. The relationships between functional disability and incontinence and between functional disability and cognition impairment were bidirectional, and functional disability played a key mediating role in the relationship between cognitive impairment and incontinence. Physical more than cognitive training in order to improve or at least stabilize functional performance could be a way to prevent or reduce the process of developing incontinence.

## Introduction

Aging society is a worldwide concern, and the size of the elderly population in Taiwan continues to increase with the development of medical technology and health care delivery systems^1^. The growth of the elderly population has triggered increasing demand for long-term care and skilled nursing institutions. Additionally, approximately 50.9% of senior citizens in need of long-term care services are veterans, and these veterans requiring care account for 23.7% of the total population of senior veterans^2^. With the growing average life span and the increasing prevalence of degenerative and psychosocial diseases with age, providing care for veterans has a considerable impact on medical resources and the cost of care.

Geriatric symptoms are often multifactorial in etiology; in addition to age-related change in physical function, risk factors such as coexisting cognitive impairment^3^, functional disability^4^, and incontinence^5^ cause or contribute to health decline. Studies have examined the association between cognitive impairment and its related factors, such as functional disability^6,7^ and incontinence^8,9^. Furthermore, other studies on incontinence^10,11^ have found that declines in cognitive^12^ and physical function^12,13^ are noteworthy contributors to incontinence but have not confirmed the direction of the association. Although most literature has confirmed that any two of these three health factors may have a bidirectional association, the pathway between the three factors and whether there is a potential moderating or mediating effect between these three is unclear.

In order to improve the effectiveness of treatment, it is necessary to develop multimodal interventions, but first we need to understand the pathways between the health factors that affect aging. Therefore, this study decided to explore all possible relationships between cognitive impairment, disability, and incontinence to find out the aging pathways of veteran residents, and to determine the directional correlation between the three health factors through path analysis. This study explored the pathway sequence *X* → *M* → *Y* among the three domains and hypothesized that *X* (independent variable) has either a direct effect on *Y* (dependent variable) in addition to an indirect effect through *M* (mediator) or an indirect effect on *Y* through *M*. The objectives of the current study, therefore, were to explore (a) the direct effects of *X* on *Y*, (b) the interaction effect of *X*_1_* *X*_2_ on *Y*, and (c) the indirect effect of *X* on *Y* through *M*. Rather than focusing on only one directional effect, we adopted the exploratory analytical approach to examine all possible pathways.

## Methods

### Study design, settings, and participants

This study performed secondary data analyses of a data set obtained from a cross-sectional study. The original study was based on one veterans’ home (VH) in Tainan, belonging to one of the tertiary veterans’ general hospital; Kaohsiung Veterans’ General Hospital, located in southern Taiwan. At the end of 2012, a total of 400 veterans were receiving personal care in VH in Tainan. All male residents living Tainan VH were invited for study and participants were enrolled if they agreed to participate. Those who were <aged 50, had altered consciousness, severe impairment in hearing or vision, and communication difficulties (N = 200) were excluded for study. To avoid interrater variability, one well-trained nurse collected the data between August and September 2013. In total, 200 residents 50 years of age or older were selected by a purposive sampling method; among them, 56 residents were excluded because of poor cognitive function or unwillingness to participate in this study, and 144 residents were interviewed (72%). This study was approved by the Human Experiment and Ethics Committee of Kaohsiung Veterans General Hospital and adhered to the principles of the Declaration of Helsinki. Residents were interviewed after their informed consent was obtained (IRB No.: VGHKS13-CT6-03).

## Measurements

### Outcome variables: cognitive impairment, functional disability, and incontinence

Cognitive function was measured using the 10-item Short Portable Mental Status Questionnaire; scores ranged from 0 to 10, with a higher score indicating poorer intellectual function^14^. Patients who made two errors or fewer were deemed cognitively intact, and those who made three or four errors were regarded as having mild cognitive impairment. Moderate impairment was defined as making five to seven errors, and severe impairment was defined as making eight to ten errors; the maximum number of errors possible was 10. Adjustments were made to scores regarding education: participants with less than a high school education were permitted one additional error, and those with more than a high school education were permitted one fewer errors^14^.

Measures of functional disability reflect limitations in performing independent living tasks, typically contain activities of daily living (ADLs) or instrumental activities of daily living (IADLs). A Chinese-translated version^15^ of the Barthel Activities of Daily Living (ADL) index^16^ was used to assess the current level of a participant’s ability for each of 10 items: feeding(10 points), grooming(5 points), toilet use(10 points), bathing(5 points), dressing(10 points), bowels(10 points), bladder(10 points), mobility(15 points), transfer(15 points), and stair climbing(10 points). Each activity was given a score corresponding to 2–4 levels of dependency ranging from 0 (unable to perform a task), 5, 10, to 15 (fully independent)^17^. Possible total scores ranged from 0 to 100 and were obtained by summing the points for each item, with higher scores indicating greater independence. In accordance with relevant studies, functional disability was categorized into five levels: fully independent (a score of 100), slight dependency (scores of 91–99), moderate dependency (scores of 61–90), severe dependency (scores of 21–60) and total dependency (scores of 0–20).

Incontinence data were determined from study subjects’ response to two questions regarding the veterans: “Does the resident have urinary incontinence?” and “Does the resident have fecal incontinence?” Incontinence was determined on the basis of an affirmative response (yes) to these questions, and incontinence severity was categorized into four levels according to responses (yes or no) to questions regarding the presence of the following conditions: regular urination and bowel movements, urinary incontinence only, fecal incontinence only, and urinary and fecal incontinence.

### Control variables

Control factors were selected on the basis of measures of objective health in the study. Potential risk factors included demographic factors, comorbidities, and medication use. Participants’ age, education level, and marital status were recorded as demographic data. Physical disease was evaluated according to comorbidity of the 10 most common chronic diseases: hypertension, heart disease, urinary disease, diabetes, gastrointestinal disease, psychiatric symptoms, dementia, stroke, rheumatoid arthritis, and malignant tumors. Medication use was classified into four levels according to the number of medications patients regularly used among the 10 most common medications (zero, one, two, three, or four or more).

### Statistical analysis

Descriptive statistics (frequencies and percentages) were determined for demographic characteristics, comorbidities, medication use, and health status. In addition, path analysis was adopted to examine the hypothesized interrelationships among cognitive impairment, functional disability, and incontinence; outcome variables were selected while controlling for important covariates^18,19^.

**Figure 1:**
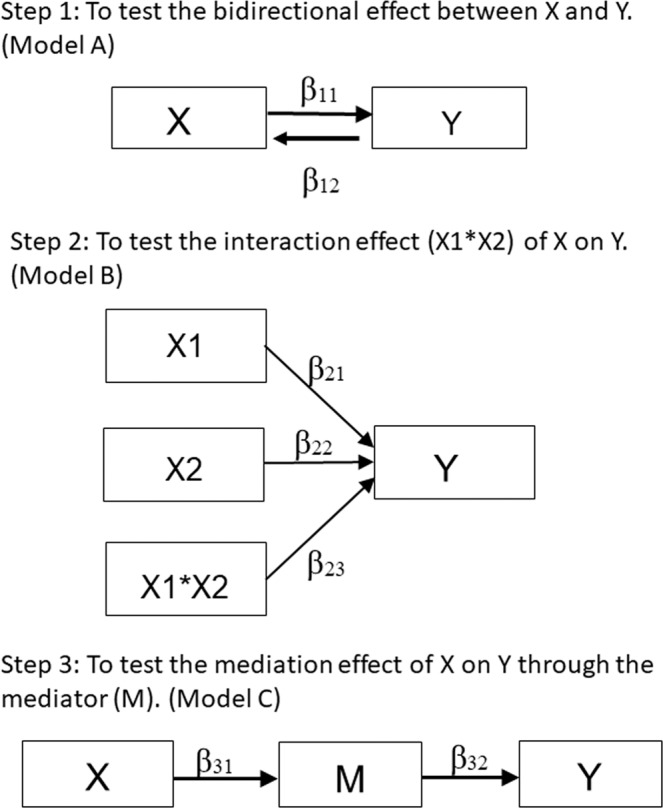
Research design and procedure of the exploratory model. Path diagram for testing direct and indirect effects (note: X = independent variable, Y = dependent variable, and M = mediating variable). The testing procedure scheme followed three steps. Complete mediation model illustrating that all of the relationships between X and Y are transmitted through the mediator (disability).

This study adopted a mediation model to test whether the relationship between *X* (Independent variable) and *Y* (dependent variable) is transmitted through the mediator (*M*) (Fig. 1). *X*, *M*, and *Y* represent cognitive impairment, functional disability, and incontinence, respectively. In doing so, this study made three assumptions: (a) *X* and *Y* have bidirectional effect (b) *X* has a direct effect on *Y* in addition to an indirect effect through *M* and (c) *X* has an indirect effect on *Y* through *M*. According to the research hypotheses, the following were examined: (a) the direct predictive effect of independent variable (*X*) on dependent variable (*Y*), (b) the interaction effect of independent variables (*X*_1_ * *X*_2_) on dependent variable (*Y*), and (c) the indirect effect of independent variable (*X*) on dependent variable (*Y*) through mediator (*M*).

For each model, demographic factors, comorbidities, and medication use were adjusted. To test the mediating processes, the bootstrap method was adopted to test direct and indirect effects for the structural models^20^. Goodness of fit was determined according to the value of chi-square value (CMIN/DF ≦ 3), comparative fit index (CFI ≧ 0.90), Tucker–Lewis index (TLI ≧ 0.90), root mean square error of approximation (RMSEA < 0.08), and p-value for GOF > 0.05, as recommended by Hu and Bentler^21^ and McDonald^22^. Furthermore, parameter values were estimated with a maximum likelihood model using IBM AMOS and data were analyzed using Statistical Package for the Social Sciences (SPSS)22.0 (IBM Corp, Armonk, NY, USA).

## Results

A total of 144 participants completed the questionnaire and were included in the final analysis. The patients’ distribution of all study variables is summarized in Table 1. Approximately 80% of the patients were aged 81 or older, 40% were married, only 9% had no formal education, and less than 8% were not taken medication. The majority of veterans were diagnosed with at least one comorbid disease (97.2%). In addition, approximately 42.4% had a functional disability, 27.8% had cognitive impairment, and 22.2% had incontinence.

**Table 1:** Characteristics of the Study Sample (N = 144).

		Frequency	Percent
Age	50–80 years old	29	20.1
	≧81 years old	115	79.9
Marital Status	Unmarried	48	33.3
	Married	58	40.3
	Widowed & divorce	38	26.4
Education	Illiterate	13	9.0
	1–9 years	49	34.0
	≧10 years	82	56.9
Number of	zero	11	7.6
Medication use	one	35	24.3
	two	34	23.6
	three	30	20.8
	four or more	34	23.6
No. of comorbidities	None	4	2.8
	1 disease	32	22.2
	2 diseases	49	34.0
	3 diseases	29	20.1
	≧4 diseases	30	20.8
Physical disability	Fully independent	83	57.6
	Mild disability	6	4.2
	Moderate disability	27	18.8
	Severe disability	18	12.5
	Total dependent	10	6.9
Cognitive status	Normal	104	72.2
	Mild impairment	26	18.1
	Moderate impairment	9	6.3
	Severe impairment	5	3.5
Incontinence	Continence	112	77.8
	Urinary only	7	4.9
	Fecal only	4	2.8
	Urinary& Fecal	21	14.6

### Pathway model estimation

Figure 1 presents the conceptual model. First, we test a hypothesized bidirectional effect between X and Y (β11&β12, β13&β14, β15&β16) (Model A). Second, the interaction effect between X1 and X2 was further test with Y (β23, β26, β29) (Model B). Third, we verify the mediating effect between X1 and X2 with Y, to find out how X affects Y through M (mediation variables) (β31& β32, β33& β34) (Model C). Within the associations among cognitive impairment, functional disability, and incontinence, the potential associated factors were also adjusted. Table 2 presents the three models with 19 path diagrams, in addition to the interaction effect, the regression coefficients for disability consistently showed a significant association with incontinence and cognitive impairment.

**Table 2:** Verification of the bidirectional, Interaction and Mediation Effects of Cognitive Impairment, Functional Disability, and Incontinence.

		X	M	Y	Estimate	S.E.	C.R.	P^#^	CMIN/DF	CFI	TLI	RMSEA	P^§^
**Model A: (Verification of Bidirectional Effect)**													
A1	β11	Cognition	−	Incontinence	0.107	0.117	1.345	0.179	1.857	0.893	0.799	0.077	0.062
A2	β12	Incontinence	−	Cognition	0.113	0.059	1.345	0.179	1.260	0.956	0.927	0.043	0.253
A3	β13	Disability	−	Incontinence	0.746	0.044	13.682	<0.001	1.017	0.999	0.998	0.011	0.432
A4	β14	Incontinence	−	Disability	0.652	0.070	11.852	<0.001	1.578	0.982	0.955	0.064	0.149
A5	β15	Cognition	−	Disability	0.187	0.138	2.466	0.014	1.646	0.951	0.896	0.067	0.117
A6	β16	Disability	−	Cognition	0.214	0.049	2.466	0.014	1.089	0.989	0.981	0.025	0.364
**Model B: (Verification of Interaction Effect)**													
B1	β21	Disability	−	Incontinence	0.745	0.055	11.125	<0.001	1.205	0.984	0.976	0.038	0.189
	β22	Cognition	−	Incontinence	−0.13	0.147	−1.269	0.204					
	β23	Disability*Cognition	−	Incontinence	0.091	0.065	0.773	0.439					
B2	β24	Cognition	−	Disability	0.255	0.166	2.790	0.005	1.442	0.979	0.957	0.056	0.082
	β25	Incontinence	−	Disability	0.729	0.087	10.596	<0.001					
	β26	Cognition*Incontinence	−	Disability	−0.146	0.078	−1.395	0.163					
B3	β27	Incontinence	−	Cognition	0.082	0.193	0.294	0.769	2.443	0.994	0.956	0.100	0.062
	β28	Disability	−	Cognition	0.416	0.109	2.122	0.034					
	β29	Incontinence*Disability	−	Cognition	−0.298	0.060	−0.773	0.440					
**Model C: (Verification of Mediation Effect)**													
C1	β31	Cognition	Disability	−	0.176	0.137	2.329	0.020	1.371	0.979	0.964	0.051	0.171
	β32	−	Disability	Incontinence	0.752	0.044	13.682	<0.001					
C2	β33	Incontinence	Disability	−	0.648	0.070	11.679	<0.001	1.584	0.977	0.946	0.064	0.113
	β34	−	Disability	Cognition	0.314	0.044	3.986	<0.001					

Cut-off for good fit: chi-square (CMIN/DF) ≦ 3, CFI ≥ 0.90, TLI ≥ 0.90, RMSEA < 0.08, p value > 0.05

P^#^: P-value for estimates.

P^§^: P-value for goodness-of-fit.

### Bidirectional effect model (Model A)

We constructed six models (A1–A6) based on different combinations of three outcome variables and tested the effect between *X* and *Y* (*X* ↹ Y bidirectional association). The results revealed that there were significant effects between functional disability and incontinence (in A3 and A4), followed by cognitive impairment and functional disability (in A5 and A6). In addition to cognitive impairment and incontinence, these findings indicate that these outcome variables affect each other, in particular a relatively strong bidirectional association between functional disability and incontinence with very significant levels (Table 2).

### Interaction effect model (Model B)

We further tested the interaction effect of *X*_2_ on the relationship between *X*_1_ and *Y*. *X*_2_ is a moderator variable if an interaction exists between *X*_1_ (predictor or independent variable) and *X*_2_ (moderating variable) that affects *Y*. The results of model B (B1-B3) revealed that the interactions were not significant (β23, β26, β29) (*p* > 0.05); the effects only existed between disability to incontinence (β21 in B1), cognitive impairment to functional disability (β24 in B2), incontinence to functional disability (β25 in B2), and disability to cognitive impairment (β28 in B3). The results of this analysis did not indicate the existence of a moderating effect (Table 2).

### Mediation effect model (Model C)

Model A and Model B have demonstrated no direct or moderating link between cognition and incontinence. To improve the model, we further tested the mediating effect in model C (C1-C2), and the results provide evidence of complete mediation (*X* → *M* → *Y*): cognitive impairment affected functional disability (β31), which in turn affected incontinence (β32) (in C1); incontinence affected functional disability (β33), which in turn affected cognitive impairment (β34) (in C2) (Table 2). The best model fit was found in the mediation model C1, adjusting for the mediation effect between incontinence to disability (Model fit: Chi-square = 1.371). The strong effect was found between functional disability to incontinence (β32 = 0.752), followed by incontinence to functional disability (β33 = 0.648), disability to cognitive impairment (β34 = 0.314), and cognitive impairment to disability (β31 = 0.176). Accordingly, disability can not only independently and significantly affect cognitive impairment and incontinence, also through cognitive impairment (incontinence) to affect incontinence (cognitive impairment). This suggests that all of the relationships between *X* and *Y* are transmitted through the mediator (Table 2).

## Discussion

The aim of this study was to examine the interrelationship among cognitive impairment, functional disability, and incontinence by using a path analysis model. Our findings indicate that older patients’ health after institution is a complex process, with three domains (cognitive impairment, functional disability, and incontinence) often simultaneously influencing each other. Two points are of interest. First, bidirectional effects have been demonstrated in disability-cognitive impairment and disability-incontinence. Second, cognitive impairment correlated with incontinence may mediate through the functional disability.

Our research findings were consistent with those of other studies that have found bidirectional relationships between cognitive impairment and functional disability^6,7,23^. Furthermore, a meta-analysis study^24^ found that physical functioning was a more consistent predictor of cognitive change than cognitive change was of physical functioning; this is in agreement with the findings of our study because stronger impacts were identified in most models. The strong association between functional disability and cognitive impairment may be unsurprising, given that functional disability is required to make a diagnosis of dementia. The findings suggest that early detection of functional disability is crucial, and physical activity is recommended for older adults with normal cognition to reduce the risk of cognitive decline.

An association between functional disability and incontinence among older people was previously described^11,25,26^. In fact, awareness of greater functional disability may cause incontinence as a physiological reaction to the loss of functioning. Studies have shown that decline in physical function, particularly impairment of mobility and lower body strength, is an important risk factor for the development of urinary incontinence frequency^27,28^. This association can be a direct consequence of the inability of getting to the toilet and removing clothes before losing urine. Thus, it is possible that the relationship between physical performance and incontinence is bidirectional^29^, generating a vicious cycle where the reduction of physical performance causes an increase in cases of incontinence and incontinence causes a reduction in physical performance. Strategies such as prompted voiding and individual physical training have been shown to have a positive effect to reduce the frequency of incontinence episodes and improve mobility endurance^25,30,31^.

In our data, the effect of cognitive impairment on incontinence was mainly explained by the significant mediator of functional disability. A cross-sectional study showed that more severe urinary incontinence (UI) was associated with more ADL impairment, and nursing home residents with UI had poor cognitive impairments, and a higher incidence of urinary tract infections^32^. As deteriorated functional disability may be an early sign rather than other risk factor for incontinence, the temporal relation between functional disability and incontinence in older veterans needs to be further tested. Nevertheless, the presence of cognitive impairment may negatively impact older patients’ subsequent functional disability and incontinence by decreasing their motivation to maintain healthy and self-care behaviors such as physical rehabilitation and engaging in role activities. Treating one condition might improve another so targeting this triad with a multimodal intervention along with necessary pharmacological therapy might not only more effectively treat incontinence but also improve overall function and cognition in older adults following an institution for medical and long-term services.

In this study, the interaction terms were not statistically significant. Since the statistical power of tests of interaction terms is drawn not from the entire sample size but from the size of the smallest of the intersecting groups it represents. About a quarter of our samples had cognitive impairment (28%) or incontinence (22%). The non-significant results of this interaction analysis might indicate that this group is highly homogeneous. Furthermore, regardless of cognitive impairment, the proportion of incontinence is similar (10–12%), which is different from other literatures. A current study found significant differences in the urinary tract between residents with and without dementia, showing that the prevalence of UI is very high even in early dementia (64%), reaching 94% in severe dementia^33^. Additional consideration of severity of cognitive impairment and of multicollinearity between cognitive impairment and incontinence would help determine the exact contribution of a cognitive impairment to risk of incontinence.

To our knowledge, this is the first study to investigate the pathways associated with cognitive impairment, functional disability, and incontinence in male veterans. In this study, no hypothesized direction of paths among three outcome variables was specified first. This exploratory approach could be strengths to provide new information of direct and indirect effects to fill knowledge gap. The dataset for this study allowed us to explore all possible associations among all three variables of interest in order to design the best intervention or treatment for the health degradation of this group.

This study had several limitations. First, its cross-sectional design limited the interpretation of risk factor associations. Although this study approach suggests the existence of a bidirectional link, it was only able to prove direct and indirect associations and not causation. There are debates as whether cognitive impairment should affect incontinence in this model rather than the direction we have found. Clearly, all three domains have complex etiologies and are interacted and we could not rule out the reverse paths in the validation analyses. In addition, given that our study design was observational, causal interpretations in this regard are limited. Second, clinical assessments cannot be performed to identify incontinence, and because of self-reporting incontinence assessment, the disease may be underreported. In order to reduce the validity and reliability issues that self-reported data may cause, we have trained nurses to collect self-reported incontinence information, and further verified the collected data with the agency’s responsible nurse. Third, the self-reported information of medication use could be incomplete as the presence of chronic conditions. The common chronic conditions listed account for 91.7% of the total conditions, and 70% of the commonly used medications listed are for these common conditions. Considering that residents with multiple diseases use more than one medication, the “quantity of medication usage” is used in the measurement. Therefore, even if the amount of medication used by some residents may be underestimated, it should not affect the results. Finally, the generalizability of the present findings is also limited by a small sample size recruited from a single VH residence. To reduce the potential factors that may confound the interrelationship, many potential risk factors, such as multimorbidity and medication use, were adjusted in the model.

## Conclusions

This study observed that functional disability was prevalent and strongly associated with incontinence and cognitive functions. Although the direct and indirect relationships between variables are substantiated in the proposed model, in the validation analyses when the alterative models were tested, we found evidence in favor of a bidirectional correlation. In addition, these pathways suggest a mediating relationship among cognitive impairment, functional disability, and incontinence in male veterans and that preventing functional disability can improve cognitive impairment and incontinence during the post-institution phase. The findings support its mediating effect and merit further corroboration. Future studies should examine the mechanisms underlying the onset timing of each condition, the identification of which is crucial for developing interventions and exploring their optimal timing to maintain optimal physical function and reduce subsequent health decline.

## Data Availability

We were given permission to use the data from the Veterans study (Grant 102-FI-GHC-IAC-R-002) by the Human Experiment and Ethics Committee of Kaohsiung Veterans General Hospital. The datasets used for this analysis are not publicly available, as the use of data from the Veterans study requires the permission of the KVGH committee.
